# The Association of rs1670533 Polymorphism in RNF212 Gene With the Risk of Down Syndrome in Young Women 

**Published:** 2018-03

**Authors:** Fatemeh Davari-Tanha, Mahbod Kaveh, Ahmad Ebrahimi, Maryam Mirzaei, Mamak Shariat, Zahra Shahraki

**Affiliations:** 1 Department of OBS & GYN, Yas Hospital, Tehran University of Medical Sciences, Tehran, Iran; 2 Department of Neonatology, Bahrami Hospital, Tehran University of Medical Sciences, Tehran, Iran; 3 Department of Molecular Genetics, Cellular and Molecular Research Center, Institute of Endocrinology and Metabolism, Shahid Beheshti University of Medical Sciences, Tehran, Iran; 4 Materno-Fetal, Neonatal Research Center, Tehran University of Medical Sciences, Tehran, Iran

**Keywords:** Down Syndrome, RNF 212, rs1670533, Polymorphism, Pregnancy

## Abstract

**Objective:** To evaluate association between polymorphism rs1670533 in RNF212 gene with the risk of Down syndrome in young women.

**Materials and methods:** In a case control study, one hundred pregnant women were evaluated in both group. The case group consisted pregnancy with diagnosis of Down syndrome in women younger than 35 years old. The control group consisted pregnancy with normal neonate. Fifty pregnant women in each group were allocated.one hundred blood samples were collected. Genomic DNA was extracted by salting – out method and polymorphism of rs1670533 were detected by PCR.PCR products were detected on 2% agarose gel electrophoresis.

**Results:** The TTrs1670533 haplotype was present in 36% of pregnant women with Down syndrome versus 14% of normal pregnant women, (p = 0.003 e-12; CI 95%1.665-5.305, OR = 3.107); TC haplotype was present in 56% of normal pregnancy regarding of16% of pregnancy with Down syndrome (p = 4.288 e = 12; CI 95%: 0.145-0.25; OR = 0.126).

**Conclusion:** It seems that TTrs1670533 haplotype is a risk factor for pregnancy with Down syndrome in young women and TC haplotype has protective effect.

## Introduction

Down syndrome or triosomy 21 is the leading cause of chromosomal abnormality in human which is caused mental retardation (1). The extra chromosome is the result of nondisjunction in meiotic division and the origin is from maternal chromosome (2), the age of mother at the time of conception is a well-known risk factor for nondisjunction and Down syndrome (3) although there are many young women who have Down syndrome children (4). There is a long time controversy about physiologic aging or chronological aging; it means that when a young ovary loss its primordical follicle pool, it results to nondisjunction at oogenesis like the times that an old age female and ovary is prone to nondisjunction at meiosis. There are many ideas about constitutive and age related changes in hormonal environment and in oocyte and follicles, which are responsible for failure in segregation of homologues and sister chromatids at meiosis (5).

Delicate chromosome segregation during meiosis is designed by crossing –over (6) and each of sister chromatid obtains at least one crossover, although a lot of recombination sites yield non-crossovers (7). A strong regulatory mechanism of crossing-over is RNF212, which is associated with alteration in crossover rates in humans (8). The mouse RNF212 is necessary for crossing-over, it regulates mechanism that couple chromosome synapsis for formation of crossover-specific recombination complexes (9). Selective localization of RNF212 to a group of recombination loci is suggested to be a fundamental early action in the crossover designation reaction (10). The responsibility of RNF212 is stabilizing these sites for meiosis-specific recombination factors, including the MutSγ complex (MSH4-MSH5) (11). Selective stabilization of crucial recombination proteins is a essential feature of meiotic crossover regulation. Haplo insufficiency indicates that RNF212 is a confining factor for crossover control and raises the possibility that human alleles may alter the amount or stability of RNF212 and be risk factors for aneuploid situations (12).

It is showed that both male and female Rnf212 -/- mice have normal phenotype but were sterile and reduced testis size and absence of post-anaphase I cells was occurred in male Rnf212 null. Also Rnf212 -/- ovaries had normal size to those of wild type animals, and the numbers of oocytes were similar to mature animals (13). Apparently normal pachytene was occurred in sperm and oocyte nuclei of Rnf212 null mouse and fully synapsed autosomes. Although, X-Y synapsis was destabilized and crossover complexes were absent in Rnf212 -/- spermatocytes. It is suggested that Rnf212 was necessary for stabilize the meiosis-specific factors Msh4 (602105) and Tex11 (300311) (14).

Two SNPs within the RNF212 gene, rs3796619 (612041.0001) and rs1670533 (612041.0002), are found (8),that are related with inverse recombination rates in men and women. Haplotype TC was associated with high female recombination rate and low male recombination (15).

At this study we aimed to evaluate the effects of polymorphism rs1670533 in RNF212 gene on the incidence of pregnancy in young age women who have Down syndrome baby and comparing them with pregnancy with healthy baby.

## Materials

This is a case control study which is included 50 pregnant women with Down syndrome neonate as case group, and 50 pregnant women with healthy neonate as control group. Case and control mothers were recruited from prenatal clinic of a tertiary university-based hospital. An informed consent was obtained from all participants mothers. The ethic committee of Tehran university of medical sciences approved this project and the ethical code was 21626. The inclusion criteria was age < 35 and pregnancy with Down syndrome which was approved by karyotype.

5-mL whole blood samples were collected from the caes and control groups and placed into special tubes containing ethylenediaminetetraacetic acid anticoagulants material. Immediately after collection, all of the samples were stored at –20°C until use. Genomic DNA was extracted from whole blood using the salting-out method. Specific primers were designed for the TCrs1670533 and TTrs1670533 genes and Gap-polymerase chain reaction (PCR) was performed for both of the genes. The sequences of primers are shown in Table 1. The PCR reaction was carried out T1 at 0.2 micro tubes with a total volume of 15 μL containing 7.5 μL Master Mix Red (Ampliqon), 1.5 μL of TCrs1670533 and TTrs1670533 primer pairs (10 pmol of each primer), 0.8 μL of Beta-globin primers (10 pmol of each primer), 3.5 μL of sample DNA (100 ng) and 1.7 μL double distilled water. The PCR reaction was performed in 30 cycles with the following conditions. The reaction mixture for TCrs1670533 was first subjected to initial denaturation at 95°C for 5 min; 30 cycles consisting of denaturation at 94°C for 30 s, primer annealing at 59°C for 40 s and DNA extension at 72°C for 30 s; the final DNA extension was at 72°C for 3 min and the amplification conditions for TTrs1670533 were initial denaturation at 94°C for 5 min followed by 30 cycles of denaturation at 94°C for 1 min, annealing at 61°C for 45 sec, extension at 72°C for 1 min, and final extension at 72°C for 7 min. The products of the PCR amplification (TC,TT, and β-gol, 102 bp) were then subjected F1 to electrophoresis on a 2% agarose gel and visualized by ethidium bromide. β-globin (Cinagen) was used as internal control in this study (Table 1).

Data were analyzed with SPSS 19 for Windows. The χ^2^ and Fischer’s exact test were used to compare variables between groups. p < 0.05 was considered statistically significant. The odds ratios (OR) with 95% confidence intervals (CI) were calculated simultaneously as an estimate of the risk for pregnancy with down syndrome in young age women.

**Table 1:** Sequences of primers used for amplification

**Name**	**Sequence of primers(5’-3’)**
TC-F	GTCTGGACACGTGGGGAGTC
TT-F	GTCTGGACACGTGGGGAGTG
B-gol-F	GTGCACCTGACTCCTGAGGAG
B-gol-R	CCTTGATACCAACCTGAACAG

## Results

There are one hundred women in this survey aged (18-35) years old. At birth of their Down syndrome child, 50 women in case group were under 35 years old. The results of this study showed that the haplotype TCrs1670533 was the most frequent type in control group (56% in control group versus 14% in case group); the haplotype TTrs1670533 was more common in the case group (36%) versus control group (16%). The difference was statistically significant p = 0.003. The odd ratio for having a Down syndrome child during pregnancy in age < 35 years old is 2.722 for women who have TT haplotype (p = 1.41 e = 10 OR = 2.722). The odd ratio for having a healthy child during pregnancy in age < 35 years old women with TC haplotype is 0.152; in the other hand the TC allele has protective effects regarding Down syndrome regarding to the TT allele which is a risk factor for having Down syndrome child (Table 2).

## Discussion

The results of this study showed that the risk of having child with Down syndrome in women younger than 35 years old is associated with TT haplotype rs1670533. Conversely the TC haplotype rs1670533 has protective effect regarding Down syndrome. The haplotype TT has 2.7 folds risk for having child with Down syndrome but TC haplotype has protective effect regarding Down syndrome.

The association between the C677T allele and maternal risk of having Down syndrome child in Jordan was evaluated (16). They evaluated the frequencies of MTHFR C677T and A1298C polymorphisms in their country. Their results showed that the mutant variant677T is associated (x2 = 6.93, p = 0.008) with all groups of case mothers, although it was not statistically significant association except for young women (OR = 4.2 95% CI: 1.61-10.97, p = 0.003). This study supposed that allele 677T play a crucial role of delivering child affected by Down syndrome in TT (homozygous) and AT (heterozygous) states (OR = 10.35, p = 0.000) (16).

The pairing, synapsis and segregation of homologous parental chromosomes (homologs) are specific features of the meiotic program. Homologous recombination has essential roles in this concert (17). Recognition of homology and DNA strand exchange promote the pairing of homologs and their delicate connection by zipper-like structures called synaptonemal complexes (18). Finally, a subset of recombination loci create crossovers, causing in stable inter homolog connections that promote homolog bi-orientation on the spindle to promote exacte disjunction at meiosis I (19). Failure to crossover or the suboptimal location of crossovers (proximal to centromeres or telomeres) is responsible for missegration of homologs. In humans, aneuploidy is the result of meiotic errors that is a leading cause of spontaneous abortion and developmental disease (20).

This study showes RNF212 as an crucial crossover factor during mammalian meiosis and provides a new idea for the molecular interactions that underlie the differentiation of crossover and non-crossover recombination. How and when specific recombination sites are designated as having a crossover fate is unknown (21).

There are a high affinity for RNF212 binding to synaptonemal complex central region that tends to outcompete binding to MutSγ-associated recombination sites (22).

**Table 2:** Frquency of rs1670533TT, TC genotypes in case and controls

	**TC**	**CT**	**TT**	**p-value**	**Odd ratio**
Down syndrome n = 50	7(14%)	25(50%)	18(%36)	0.003	3.1
Control n = 50	28(56%)	14(28%)	8(16%)	1.234	0.126
Total = 50	35(35%)	40(40%)	25(25%)		

Authors cloned full-length mouse Rnf212. The deduced 307-amino acid protein has an N-terminal ring finger domain, followed by a coiled-coil domain and a C-terminal serine-rich domain. The ring finger domain is characteristic of E3 ligase enzymes that catalyze protein modification by ubiquitin-like molecules (23). The human RNF212 protein shares significant identity with the full-length mouse protein and has a similar domain structure. They also showed two splice variants of mouse Rnf212 that encode C-terminally truncated proteins of 133 and 52 amino acids (24). Immunohistochemical analysis of mouse spermatocyte and oocyte nuclei revealed dynamic localization of Rnf212 to synaptonemal complexes, including pseudoautosomal regions of X-Y chromosomes. Rnf212 localized more weakly to DNA double-strand break sites (25).

## Conclusion

According to the results of this study, It seems that TTrs1670533 haplotype is a risk factor for pregnancy with Down syndrome in young women and TC haplotype has protective effect.
